# Continuous intra-arterial nimodipine infusion in refractory symptomatic vasospasm after subarachnoid hemorrhage

**DOI:** 10.1186/s40064-016-3495-4

**Published:** 2016-10-18

**Authors:** Raimund Helbok, Alexandra Zangerle, Andreas Chemelli, Ronny Beer, Thomas Benke, Rainer Ehling, Marlene Fischer, Martin Sojer, Bettina Pfausler, Claudius Thome, Erich Schmutzhard

**Affiliations:** 1 Department of Neurology, Neurological Intensive Care Unit, Medical University of Innsbruck , Anichstrasse 35, 6020 Innsbruck, Austria; 2 Department of Radiology, Medical University of Innsbruck, Innsbruck, Austria; 3 Department of Neurosurgery, Medical University of Innsbruck, Innsbruck, Austria

**Keywords:** Subarachnoid hemorrhage, Vasospasm, Delayed cerebral ischemia, Nimodipine

## Abstract

**Introduction:**

Vasospasm still is a major cause of morbidity after aneurysmal subarachnoid hemorrhage. The purpose of this report is to describe the successful management of severe refractory vasospasm with continuous intra-arterial nimodipine (IAN) treatment.

**Case description:**

A 72-year old right handed woman was admitted with non-traumatic SAH WFNS grade 1. Cerebral computed tomography demonstrated thick blood filling of the basal cisterns, and intraventricular hemorrhage. Cerebral angiogram failed to detect a vascular abnormality. After an uneventful initial course the patient developed symptomatic left middle cerebral artery vasospasm with aphasia and corresponding restriction in diffusion weighted images in the left frontal lobe. Bolus IAN only transiently improved cerebral circulation and clinical signs and symptoms. Continuous-IAN was started and led to full clinical recovery and normalisation of MRI diffusion restrictions.

**Discussion and conclusions:**

Continuous selective intra-arterial infusion of nimodipine may be an option in selected patients with symptomatic vasospasm refractory to conventional treatment after careful consideration of benefits and procedure-related risks.

## Background

Despite advances in medical and endovascular therapy cerebral vasospasm still contributes substantially to the high morbidity and in patients with aneurysmal subarachnoid hemorrhage (SAH) (Diringer et al. 2011). Reduction in cerebral blood flow (CBF) and brain tissue oxygen tension (PbtO2) may result in ischemic brain damage which is commonly referred to as delayed cerebral ischemia (Dorsch 2011). Currently, established treatment strategies for cerebral vasospasm comprise prophylactic oral nimodipine treatment and induced hypertension (Kassell et al. 1982; Muench et al. 2007; Raabe et al. 2005).

Advances in endovascular therapy have offered new perspectives in the treatment of symptomatic vasospasm including transluminal balloon angioplasty (Dion et al. 1990) and pharmacological dilatation with spasmolytic drugs (Weant et al. 2010). Although efficacious, angioplasty is limited to large vessels; the effect of intra-arterial drugs is temporary and restricted to the infusion period. Continuous intra-arterial nimodipine (IAN) treatment may overcome these limitations and has been reported to be safe in symptomatic cerebral vasospasm after SAH (Wolf et al. 2010; Musahl et al. 2011; Ott et al. 2014; Doukas et al. 2011).

Here, a case with refractory symptomatic vasospasm developing delayed cerebral ischemia (DCI) successfully treated with continuous IAN and her corresponding clinical and imaging findings are presented.

## Case description

**Fig. 1:**
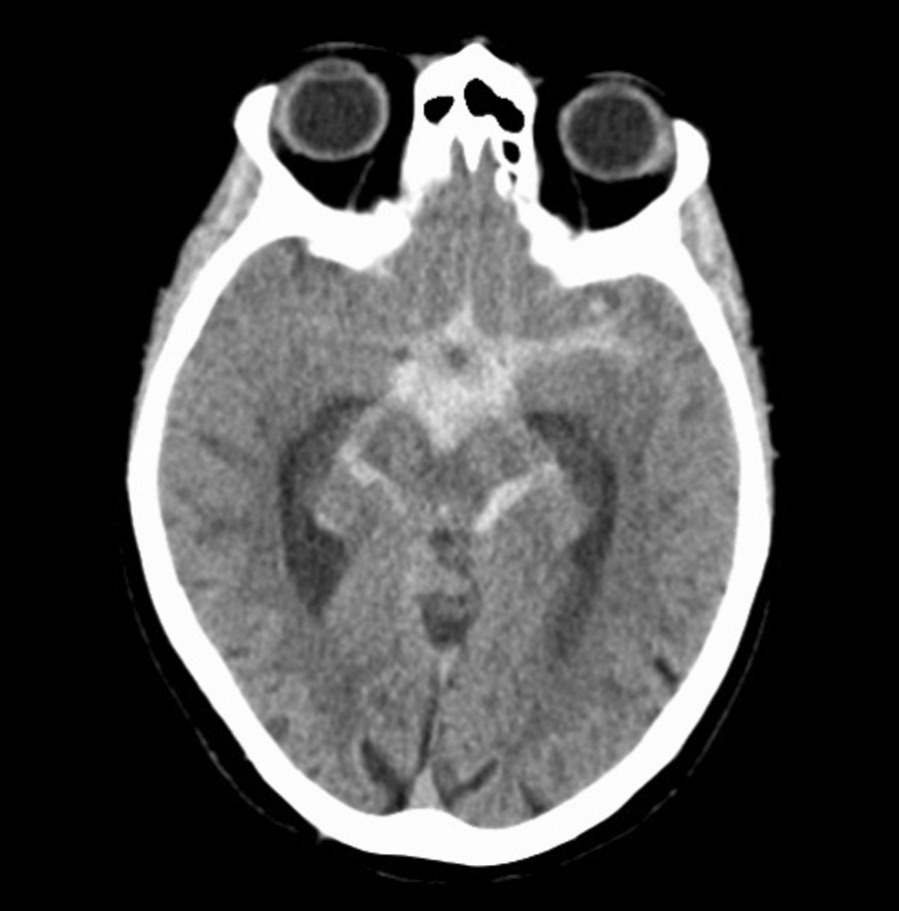
Axial head computed tomography showing subarachnoid hemorrhage with thick blood filling the basal cisterns

**Fig. 2:**
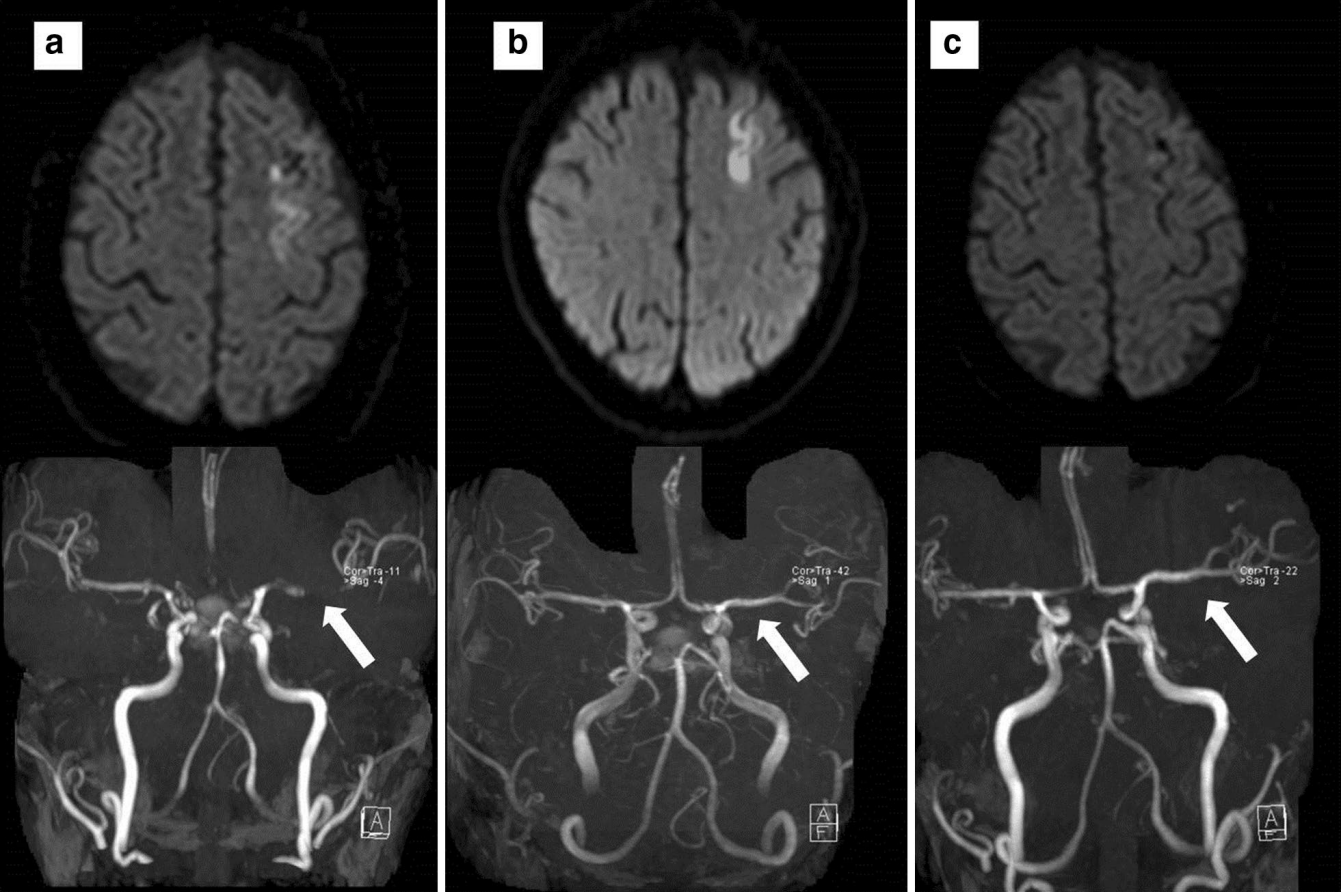
Axial magnetic resonance imaging demonstrating severe *left middle cerebral* artery vasospasm (time of flight angiography, *white arrow*) and *left frontal cortical* hyperintensities (diffusion-weighted imaging) before (**a**), 3 days after (**b**) continuous intra-arterial nimodipine treatment and at 23d follow up (**c**)

A 72 year old right handed woman presented with sudden onset of severe occipital headache and nausea. In the emergency department, neurologic examination revealed meningeal signs, without focal neurological deficits or impairment of consciousness. Head computerized tomographic (CT) scanning revealed SAH with thick blood filling of the basal cisterns and unilateral intraventricular hemorrhage (Fig. 1). Cerebral angiogram failed to detect a vascular abnormality. The patient was transferred to the Neurological Intensive Care Unit (NICU) and prophylactic intravenous nimodipine treatment was started. On day 1 her mental status declined, and head CT scanning revealed hydrocephalus, necessitating extraventricular drainage. Further hospital course was uneventful until day 10 when transcranial Doppler sonography (TCD) revealed increased mean flow velocity (140 cm/s) of the left middle cerebral artery (MCA). On day 13 the patient developed speech initiation problems and nonfluent speech. At this time TCD showed a mean flow velocity of 190 cm/s. The patient was euvolemic and despite induced hypertonia using norepinephrine (MAP increase from 87 to 98 mmHg), signs and symptoms worsened to expressive aphasia. Cerebral magnetic resonance angiography (MRA) was performed—while the angiography suite was set up—and revealed severe left MCA vasospasm and left frontal cortical and subcortical hyperintensity lesions on diffusion-weighted imaging (DWI) (Fig. 2, Panel A) without corresponding T2-hyperintensities (data not shown). Cerebral angiogram confirmed severe left MCA vasospasm and 1 mg nimodipine over 60 min was infused into the left internal carotid artery. Balloon angioplasty was not attempted. Restoration of cerebral circulation with minimal clinical improvement was observed, however the patient worsened immediately after this treatment was discontinued. The catheter was left in place in the internal carotid artery at the height of C3/C4 segment and the patient was transferred back to our NICU. At all times during the IAN infusion the patient was hemodynamically stable. Continuous infusion of IAN was now started with 0.4 mg/h and heparin anticoagulation was performed through the microcatheter targeting serum prothrombin time of 40–50 s. Blood pressure was continuously monitored and norepinephrine was titrated to maintain a MAP of over 100 mmHg. The patient gradually improved over the next 24 h and IAN was again discontinued, with the microcatheter left in place. As the patient immediately redeveloped focal signs and symptoms, treatment was restarted and continued for another 20 h leading to full recovery. As after discontinuation of IAN the patient remained now asymptomatic repeated angiogram was not performed and the microcatheter was removed without complications. The induced hypertensive treatment was slowly titrated to a MAP of 70 mmHg when norepinephrine was finally stopped. MCA vasospasm fully resolved (MRA, Fig. 2b) and MRI-DWI hyperintensities slightly improved. Follow up angiography on day 23 revealed complete resolution of cerebral vasospasm and no aneurysm was detected. The patient was discharged for neurorehabilitation with minimal deficits in fluent speech. Follow-up head-MRI on day 40 showed no structural abnormality in DWI (Fig. 2c) and T2 sequences (data not shown). The patient fully recovered when she was last seen at follow up at 3 months.

## Discussion and evaluation

Here we describe a favorable clinical course of a non-traumatic SAH-patient with refractory vasospasm successfully treated with selective continuous intra-arterial nimodipine.

Cerebral vasospasm still contributes to high mortality and morbidity after SAH (Dorsch 2011). Currently recommended treatments for vasospasm are limited to prophylactic oral or intravenous nimodipine (Dorhout Mees et al. 2007), permissive hypertension (Kassell et al. 1982), and balloon angioplasty (Diringer et al. 2011; Dion et al. 1990). We did not perform balloon angioplasty in our patient as this is not a routinely available procedure at our institution. Other pharmacological attempts to decrease large vessel vasospasm are potentially efficacious, however did not translate into improved outcome (Macdonald et al. 2011). Intra-arterial application of spasmolytic drugs resulted in transient restoration of cerebral circulation, however is limited to the infusion period (Weant et al. 2010). Endovascular continuous intra-arterial treatment may be interesting and has been shown to be safe and feasible, however should be interpreted with caution due to limited experience (Wolf et al. 2010; Musahl et al. 2011; Doukas et al. 2011). The concept of continuous application is supported by the selective treatment of the affected vessels, the possibility to treat large and small vessel vasospasm and the prolonged effect. Continuous selective IAN has been successfully administered at a dose between 0.4 and 2 mg/h (Wolf et al. 2010; Musahl et al. 2011; Ott et al. 2014; Doukas et al. 2011). No significant hypotension, which could decrease perfusion and worsen ischemia, occurred in those patients. Potentially associated risks like dislocation of the angiography catheter, catheter-thrombosis and brain damage secondary to the alcoholic solution of nimodipine did not occur, neither in our patient, nor in patients of those case series. Dilatation of spastic arteries starts within a few hours and is long-lasting. A treatment course of 44 h was safe in our patient, however may be increased to up to 5 days as shown by previous reports (Wolf et al. 2010; Musahl et al. 2011).

A concern of intra-arterial vasodilator therapy is a secondary increase in intracranial pressure (ICP) associated with a decrease in brain tissue oxygenation. Although we did not measure brain tissue oxygen tension (P_bt_O_2_) in our patient, clinical improvement did not suggest this nor a rise in ICP. In unconscious patients multimodal neuromonitoring devices measuring P_bt_O_2_, local cerebral blood flow and ICP may be helpful to monitor IAN treatment (Musahl et al. 2011). In a series of 30 patients with refractory vasospasm a prompt increase in cerebral blood flow was observed (Ott et al. 2014).

Our patient had developed effortful, slow and non-fluent speech associated with MRI-DWI hyperintensities in the cortical and subcortical left frontal region, an area functionally related to speech motor control (Ackermann et al. 1996). Signs and symptoms redeveloped when IAN was discontinued suggesting a direct effect of IAN on intracranial hemodynamics. So far, IAN cannot be recommended as routine treatment for refractory cerebral vasospasm after SAH, however may be added to endovascular treatment options (Ott et al. 2014). One important limiting factor is the need for systemic heparinization and the possible risk of recurrent intracranial hemorrhage, particularly in patients with presumed ruptured aneurysms that had not yet been identified or secured.

## Conclusions

Continuous selective intra-arterial infusion of nimodipine may be considered in selected patients with symptomatic high grade vasospasm refractory to standard treatment including angioplasty. The potential benefits of IAN should be balanced with procedure-related risks in symptomatic refractory vasospasm after SAH.

## Abbreviations

CT: computed tomography
DWI: diffusion-weighted imaging
IAN: intra-arterial nimodipine
ICP: intracranial pressure
MCA: middle cerebral artery
NICU: neurological intensive care unit
P_bt_O_2_: brain tissue oxygen tension
SAH: subarachnoid hemorrhage
TCD: transcranial doppler
